# Healthcare inequalities in general practice due to educational level: A retrospective cohort study analysing patients’ presentation and GP response to requests

**DOI:** 10.1080/13814788.2026.2649992

**Published:** 2026-04-14

**Authors:** Sjoerd Hulshof, Tim C. olde Hartman, Reinier Akkermans, Henk J. Schers, Annemarie A. Uijen

**Affiliations:** Department of Primary and Community Care, Radboud University Medical Center Nijmegen, Nijmegen, The Netherlands

**Keywords:** Educational status, General Practice, reason for encounter, diagnostic and therapeutic interventions

## Abstract

**Background:**

Educational level is an important social determinant of health and may contribute to healthcare inequity by affecting how patients present health problems.

**Objectives:**

Explore the interaction between patients’ educational level (1), their presentation of health problems to general practitioners (GPs) and (2) GP’s responses to requested interventions.

**Design and setting:**

Retrospective cohort study within a Dutch primary care practice-based research network.

**Methods:**

All new episodes of care of patients’ aged ≥25 years between 2014 and 2022 were included. Data were collected on the reason for encounter (RFE) at initial contact within each episode, and patients’ educational level. Differences were analysed in incidence of RFE types (symptoms, intervention-requests, self-diagnoses) and GP’s policies regarding requested diagnostic and therapeutic interventions among patients with low, medium and high educational levels.

**Results:**

Patients with lower educational levels more frequently presented symptoms (59.7% versus 56.5%) and were less likely to present with intervention requests (OR 0.88) or self-diagnosis (OR 0.83). They requested more urine tests (RR 1.28), but fewer blood tests (RR 0.90), diagnostic imaging (RR 0.75) and referrals to primary (RR 0.74) and secondary care (RR 0.87). GPs responded more often to urine test requests (RR 1.25), but less often to referral requests to primary (RR 0.68) and secondary care (RR 0.80) among patients with lower educational levels.

**Conclusion:**

This study emphasises GPs’ need to understand how educational status affects patient’s presentation and intervention preferences, which can improve communication, shared decision-making and enhance equitable healthcare delivery by addressing an important social determinant of health.

## Introduction

Inequities in health exist both within and between countries, arising from social determinants of health such as healthcare access, economic stability and education access [1–3]. These factors influence how patients perceive and present health problems [2]. Research indicates that consultation frequency is higher among older adults, women, immigrants, those with chronic conditions and individuals experiencing psychological distress, while physical activity and higher educational levels are linked to lower consultation rates [4–7]. Higher education is also associated with longer consultations, fewer medical tests and lower reporting of psychosocial issues [8,9].

Social determinants similarly impact GPs’ diagnostic and therapeutic decisions. Patients with higher socioeconomic status are less likely to receive prescriptions but more likely to be referred to specialists [10,11]. Women receive fewer physical examinations and specialist referrals but undergo more laboratories testing than men [12]. Among socioeconomic factors, educational status is the strongest predictor of healthcare utilisation [13,14].

Recognising that patients presentation styles vary; symptom-based (e.g. fatigue), self-diagnosis (e.g. ‘I have the flu’) or direct intervention requests (e.g. ‘I need a blood test’) is crucial for GPs to mitigate inequities. It helps GPs to understand patients’ needs more accurately and communicate effectively. This ensures equitable care by reducing misunderstandings and supporting shared decision-making. As far as we know, no research has been done on how educational status effects the different ways in which a patient presents to their GP. Examining the impact of educational status on primary care contributes to a more nuanced understanding of the influence of one of the social determinants of health on health inequalities. It allows GPs to understand and potentially minimise health inequities due to patient’s educational status. Additionally, little research is done on the effect of educational status and the likelihood of a GP adhering to a patients’ request.

To address this gap in the literature, this study will explore the association between patients’ educational status and the way in which patients present a health problem to the GP (as a symptom, a request for an intervention, or a self-diagnosis). Additionally, we will examine the effect of educational level on the response of GPs to patients’ requested interventions.

## Methods

### Design and setting

We conducted a retrospective cohort study using data from the Family Medicine Network (FaMe-Net), a practice-based research network affiliated with Radboud University Medical Centre, Nijmegen, the Netherlands [15,16]. As of 2022, FaMe-Net comprises 35 GPs across six practices, serving approximately 43,000 patients.

FaMe-Net systematically records medical data within episodes of care, encompassing all consultations and interventions related to a new health problem. An episode of care is defined as a new health problem presented by a patient and all consultations and interventions related to this health problem. The reason for encounter (RFE) reflects a patient’s demand for care and, together with diagnoses and interventions, is coded using the World Health Organisation International Classification of Primary Care, 2nd edition (ICPC-2) [17]. According to the ICPC-2 classification, RFEs are categorised as a symptom (codes 1–29, e.g. dysuria), a request for intervention (codes 30–69, e.g. urine test), or a self-diagnosis (codes 70–99, e.g. cystitis). Coding of the RFE, diagnosis and performed interventions is done by GPs during all types of consultations, including face-to-face, phone, email and online encounters.

The validity of the data registration is high due to regular meetings among participating GPs to discuss the diagnostic criteria, which reduces registration bias. Additionally, the automated GP information system detects inconsistencies in the registration process.

Since 2016, FaMe-Net has systematically collected demographic data from adults (>18 years) *via* patient questionnaires. These questionnaires, developed through literature review and focus groups, are emailed at enrolment and biannually to newly enlisted patients. Patients were asked to complete the questionnaire once. Collected data includes highest educational level and country of birth (patient and parents).

### Patients

We included patients all patients that were registered in one of the FaMe-Net practices during the study period 1 January 2014 until 31 December 2022 and who were ≥25 years old at the time of completing the questionnaire. Patients were excluded if data on education or country of birth were missing, if they were <25 years at the time of completing the questionnaire (assuming that educational attainment is largely stable beyond this age) or if they had been registered for <365 days to ensure reliable consultation rate data.

### Data collection

We analysed the RFE in all first consultations within a new episode of care. An episode of care may start with more than one RFE (e.g. ‘I have a cough and shortness of breath’). All RFEs recorded at the start of the episode were included in the analysis. Patient characteristics included age at end of study period, sex, education level, chronic conditions, country of birth (patient and parents) and yearly consultations. Chronic conditions were defined as persistent diseases (lasting 6 months or longer) requiring healthcare attention (see Supplemental Appendix 1) [18,19]. Consultations included home visits, in-person, phone and e-consultations.

RFEs were categorised as symptoms (ICPC 1–29), intervention requests (ICPC 30–69) or self-diagnoses (ICPC 70–99). The six most frequently mentioned intervention requests were analysed: blood test (ICPC 34), urine test (ICPC 35), diagnostic imaging (ICPC 41), medication request (ICPC 50), primary care referral (ICPC 66) and secondary care referral (ICPC 67). To assess GP response, we analysed all interventions that were explicitly requested by the patient, and examined whether these were subsequently performed by the GP during the same consultation.

Educational status was classified into low, medium, or high based on Dutch national standards [20]. Immigration status was defined as a patient born outside the Netherlands or having at least one parent born abroad.

### Data analysis

We conducted statistical analyses using IBM SPSS Statistics 29.0. Descriptive analyses summarised patient characteristics across education levels, using means and confidence interval for continuous variables and frequencies and percentages for categorical variables. Chi-square tests assessed differences in sex, multimorbidity and immigration status, while One-Way ANOVA tested age and yearly consultations across education levels.

To examine differences in RFE by education level, we performed multinomial logistic regression, with symptoms as the reference category and higher educational level as the reference group. This resulted in odds ratios for RFEs across education levels.

For secondary analyses, we evaluated GP response to intervention requests using multilevel Poisson regression, comparing patients with high versus medium or low educational levels. For each intervention request, multilevel Poisson regression generated rate ratios for requested and performed interventions across education levels. The regression analysis has been adjusted for potential confounding factors: age, sex, multimorbidity (the presence of two or more chronic health conditions), immigration status, the number of consultations per year and the patients’ GP office. A p-value of < 0.05 was considered to be statistically significant, based on two sided tests.

## Results

### Included patients

**Figure 1.**
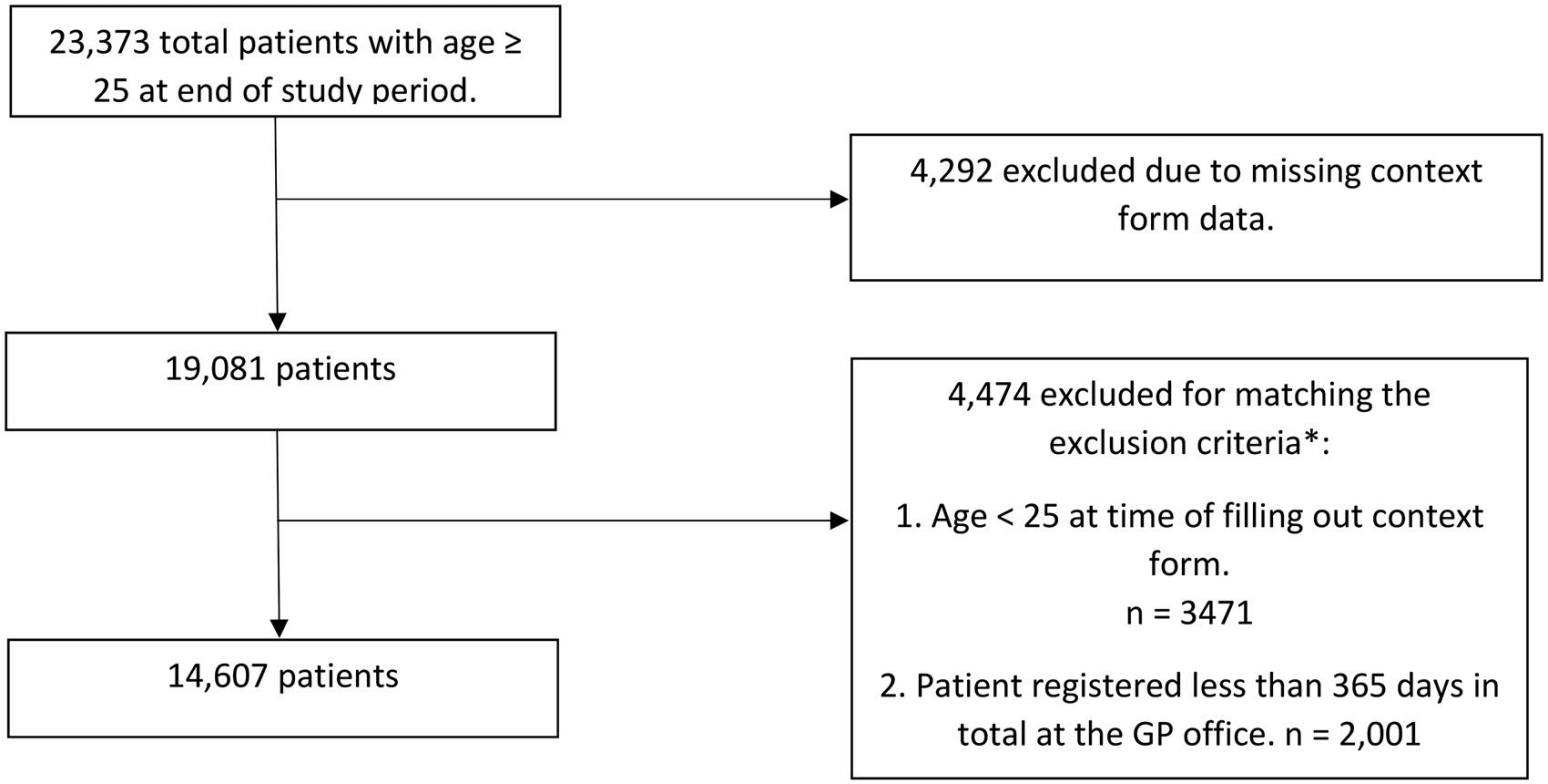
Flowchart patient exclusion.

Of 23,373 patients (≥25 years) registered during the study period, 19,081 had questionnaire data. After exclusions, 14,607 patients remained, accounting for 177,863 new episodes of care and 198,216 RFEs (Figure 1). Table 1 compares characteristics of the total population and included patients.

**Table 1.** Comparison between total study population (>25 years of age) and included patients.

	Total population	
	(>25 years of age)	Included patients
	(*n* = 23,373)	(*n* = 14,607)
Male sex (%)	48.4	45.7
Age at end of study period (mean)	43.6	41.0
Consultations per year (mean)	6.5	6.9
Reason for encounter (RFE) (%)		
*Symptom*	56.9	57.7
*Intervention-request*	29.3	28.2
*Self-diagnosed disease*	13.8	14.2

Symptom: ICPC-codes 1–29; Intervention-request: ICPC code 30–69; Self-diagnosed disease: ICPC-code 70–99.

#### Patient characteristics

Table 2 shows baseline characteristics by education level. The mean age was 41.0 years and 45.7% were male. All demographics differed significantly across education groups. Patients with lower educational levels were older, had higher multimorbidity rates and more annual consultations than patients with higher educational levels.

**Table 2.** Patient characteristics and type of RFE stratified by level of education.

*Characteristics*	Low educational level (*n* = 1751)	Medium educational level (*n* = 3801)	High educational level (*n* = 9055)	*p*-Value
Male %	45.3	48.0	44.8	<0.01
Mean Age (95% CI)	49.3 (48.5–50.0)	41.4 (41.0–41.9)	37.0 (36.7–37.3)	<0.01^b^
Multimorbidity %	86.0	80.2	70.1	<0.01
Immigration status %	23.6	21.9	18.4	<0.01
Consultations per year (95% CI)	8.9 (2.3–35.3)	6.9 (1.8–27.4)	5.4 (1.4–21.4)	<0.01
*RFE*				Total % (*n*)
Symptom % (*n*)	59.7 (17,939)	59.1 (32,695)	56.5 (63,723)	57.7 (114,357)
Intervention request % (*n*)	28.7 (8630)	27.9 (15,419)	28.1 (31,713)	28.1 (55,762)
Self-diagnosed disease % (*n*)	11.6 (3489)	13.1 (7243)	15.4 (17,365)	14.2 (28,097)
Total % (*n*)	100 (30,058)	100 (55,357)	100 (112,801)	100 (198,216)

RFE: Reason for Encounter.

CI: Confidence Interval.

Symptom: ICPC-codes 1–29; Intervention-request: ICPC code 30–69; Self-diagnosed disease: ICPC-code 70–99.

### Relation between type of RFE and educational status

We analysed 198,216 RFE types to examine their distribution across education levels. Patients with lower educational levels more often presented with symptoms and less with self-diagnoses than patients with higher educational levels (Table 3). Patients with low or medium educational levels were significantly less likely to present with an intervention request (OR respectively 0.88, 0.94) or self-diagnosis (OR respectively 0.83, 0.87) than patients with higher educational levels compared to a symptom RFE (Table 3).

**Table 3.** Type of RFE by educational status.

		Level of education		
		Low	Medium	High
RFE category	Intervention request (OR, 95% CI)	0.88 (0.85–0.91)	0.94 (0.91–0.96)	1
	Self-diagnosis (OR, 95% CI)	0.83 (0.79–0.87)	0.87 (0.84–0.90)	1

The reference category for RFE category is symptom.

RFE: Reason for encounter; OR: Odds Ratio; CI: Confidence Interval.

Intervention-request: ICPC code 30–69; Self-diagnosed disease: ICPC-code 70–99.

### Requested interventions and GP response to requests

Patients with lower educational levels requested blood tests less often than patients with higher educational levels (RR 0.90, p-value 0.03), with no difference in GP response (Table 4). However, urine test requests and GP response were more frequent in patients with low and medium educational levels (RR requested respectively: 1.28, 1.18; RR performed respectively: 1.25, 1.16) (all *p*-values <0.01).

**Table 4.** Requested interventions per year and GP response to these requests across three different levels of education.

		Requested interventions		Performed interventions	
Type of intervention	Educational level	Number per 1000 patients	Rate-ratio (95% CI)	Number per 1000 patients	Rate-ratio (95% CI)
Blood test	Low	40.1	0.90 (0.82–0.99)*	20.0	0.93 (0.81–1.06)
	Medium	44.1	0.99 (0.92–1.06)	20.6	0.96 (0.86–1.06)
	High	44.5	1	21.5	1
Urine test	Low	66.2	1.28 (1.21–1.35)*	26.4	1.25 (1.15–1.36)*
	Medium	61.1	1.18 (1.13–1.23)*	24.4	1.16 (1.01–1.24)*
	High	51.9	1	21.1	1
Diagnostic radiology and imaging	Low	4.5	0.75 (0.58–0.95)*	1.8	0.68 (0.46–1.00)
	Medium	5.6	0.93 (0.78–1.12)	2.7	0.98 (0.75–1.29)
	High	6.0	1	2.7	1
Medication	Low	80.8	1.07 (1.01–1.14)*	44.7	1.02 (0.94–1.10)
	Medium	74.7	0.99 (0.94–1.04)	43.8	1.00 (0.94–1.06)
	High	75.5	1	44.0	1
Referral to other primary care provider	Low	9.9	0.74 (0.63–0.88)*	4.6	0.68 (0.54–0.85)*
	Medium	12.1	0.91 (0.81–1.01)	5.9	0.87 (0.75–1.02)
	High	13.4	1	6.8	1
Referral to secondary care provider	Low	22.7	0.87 (0.77–0.97)*	11.0	0.80 (0.68–0.94)*
	Medium	25.9	0.99 (0.91–1.07)*	12.0	0.94 (0.84–1.07)
	High	26.3	1	13.0	1
		Total % (*n*)		Total % (*n*)	
Total interventions	Low	15.6 (4929)		15.0 (2361)	
	Medium	28.4 (8973)		28.0 (4414)	
	High	56.0 (17,708)		57.1 (9015)	
	Total	100 (1601)		100 (15,790)	

CI: Confidence Interval.

* Statistically significant.

Results are adjusted for age, sex, multimorbidity (the presence of two or more chronic health conditions), immigration status, the number of consultations per year and the patients’ GP office.

Patients with lower educational levels requested fewer radiology and imaging tests (RR 0.75, *p*-value 0.02), but GP response did not differ. No significant differences were found in medication requests and GP response.

Patients with lower educational levels requested fewer referrals to primary and secondary care than patients with higher educational levels (RR respectively: 0.74, 0.87; *p*-value < 0.01), with lower GP response compared to patients with higher educational levels (RR respectively: 0.68, 0.80; *p*-value 0.01).

## Discussion

This study examined the impact of educational level on patient presentation and GP response to requested interventions. In this study, we deliberately focused on patient-requested interventions; interventions initiated solely by the GP were beyond the scope of this analysis. Patients with higher educational levels tended to express specific expectations or offer their own diagnosis, whereas patients with lower educational levels more often described general symptoms. This pattern may reflect both differences in communicative confidence and genuine variations in health literacy, including patients’ ability to recognise and articulate health problems.

Educational level influenced both requests and GP management. Patients with lower educational levels were more likely to request and receive urine tests. This may reflect underlying differences in clinical presentation. Previous research suggests that individuals with lower educational levels or lower health literacy may have a higher prevalence of infectious conditions, including urinary tract infections [21–23].

The nature of a medical request can impact GP’s response. Requests that align with perceived medical need are more likely to be followed. Enhanced health literacy may shape patients’ comprehension of diseases and their awareness of available healthcare services.

### Comparison with other studies

Healthcare disparities are profoundly shaped by social determinants of health like socioeconomic status and educational attainment [24,25]. Our findings align with this, showing that educational level affects how patients present health problems and how GPs respond to intervention requests.

Health literacy plays a key role in patient engagement. Sørensen et al. highlight that patients with higher educational levels navigate healthcare more effectively, articulate concerns clearly, and engage proactively [26]. Little et al. found that doctors’ behaviour in the consultation is most strongly associated with the doctors’ perceived medical need of the patient [27]. This may explain why urine test and referral requests saw higher GP response, while blood test and radiology requests did not.

Previous studies found that patients with lower educational levels receive more medical tests per visit [9], higher drug prescriptions [10] and less gynaecologist referrals for alarm symptoms [11]. Since we analysed only requested interventions, we cannot determine if certain groups receive more interventions overall.

The Netherlands Institute for Health Services Research (NIVEL) reported an average of 5.3 contacts per person with general practice in 2024 [28]. However, as 78.4% of the population had at least one contact; this corresponds to approximately 6.8 contacts per person among those with at least one consultation. In our study, including only patients with at least one GP contact, the average number of contacts per person was 5.4, 6.9, and 8.9 among patients with high, medium and low educational levels, respectively. Our findings are consistent with previous research indicating that individuals with lower educational attainment tend to have more frequent contacts with general practice [29,30].

### Strengths and limitations

As far as we know, this is the first study analysing how educational levels affect patients’ presentation of health problems. The study setting authentically reflects the day-to-day practices observed in primary care increasing generalisation. The study gains strength from its extensive population size, with 14,607 patients of whom we obtained data on healthcare usage and detailed data on personal and contextual characteristics. This large study population led to the inclusion of 198,216 distinct RFEs. We accounted for various variables across different educational levels, such as multimorbidity, age and immigration status, thereby improving the reliability of the outcomes. Finally, the data within the FaMe-Net database is considered comprehensive, reliable and accurate, thanks to the proactive involvement of participating GPs.

Several limitations should be considered. First, educational level was obtained through a patient questionnaire resulting in the exclusion of 4292 patients (18%) due to missing data. Nevertheless, educational information was available for 82% of eligible patients, who represents a relatively high response rate for routinely collected questionnaire data. Despite this, selection bias cannot be excluded. Although we found no clinically significant differences between the included and total population, lower socioeconomic status and immigration status may be underrepresented. The patient population is comparable to the Dutch population in terms of age and gender. However, patients with higher educational levels are overrepresented in our study (62% versus 36% nationally), which consequently results in an underrepresentation of patients with low (12% versus 26% nationally) and medium (26% versus 38% nationally) educational levels [31]. Although this limits generalisibility, the absolute number of patients with low or medium educational levels (*n* = 1751 and *n* = 3801 respectively) still provides sufficient statistical power to examine the relationship between educational level and patient’s presentation. In theory, linkage with national registry data could provide more complete information on educational level or socioeconomic status. However, this was not feasible in the present study because the routinely collected primary care data were fully anonymised and obtained using an opt-out procedure, precluding linkage with external data sources. Future studies that link primary care data with national registry information may help to overcome this limitation.

Another limitation is that the RFE captures only the patient’s initial expression of their healthcare need and does not fully reflect the content of a patient-centred consultation. The RFE should therefore be interpreted as a marker of initial presentation rather than a complete representation of patients’ underlying problems or expectations, and caution is warranted to avoid overinterpretation of its role in explaining GP decision-making.

Additionally, educational status can change over time. We minimised this by only including patients aged 25 or older, but changes can still occur. The COVID-19 pandemic may have influenced RFEs, with an increase in telephone and e-consultations and fewer face-to-face visits in 2020–2021, potentially affecting patient presentation patterns [32].

### Implications

Our results show that educational level influences both patients’ presentation and GP’s responses to requested interventions. Improving the understanding of variations in patient presentation can facilitate more effective communication by allowing the GP to adjust their communication style. For example, GPs may adopt a more exploratory and supportive approach with patients with lower educational levels. This could include actively asking about patient’s concerns, expectations and possible underlying requests. Such strategies may support shared decision-making by actively encouraging patient engagement. Ultimately, this understanding empowers GPs to allocate resources more equitably, contributing to the broader goal of reducing healthcare inequalities. Future research should explore why patients with different educational backgrounds present health problems in varying ways and whether differences in GP responses arise from clinical judgement, perceived legitimacy of the request, or from patient assertiveness and pressure. By focusing on the first consultation within an episode of care, we aimed to capture the patient-driven component of presentation and requested interventions. The first consultation most directly reflects the patient’s own preferences, whereas follow-up consultations may be influenced by prior GP advice, potentially modifying the patient’s original requests. This approach allowed us to isolate how educational status affects initial patient behaviour and GP response. Future research could extend the analysis to follow-up consultations to examine how interventions evolve over time and whether inequities persist beyond the initial encounter.

## Conclusion

Educational level influences how patients present health problems and how GPs respond to requested interventions. Higher level of education is associated with a greater likelihood of presenting with an intervention request or self-diagnoses. GPs perform more referrals to primary and secondary care in patients with higher educational levels and perform more urine tests in patients with lower educational levels. Lastly, GPs responded more often to referral requests among patients with higher and to urine test requests among patients with lower educational levels. Understanding these disparities can enhance communication, promote shared decision-making and improve resource allocation.

## Supplementary Material

Supplemental Material
