# Supplementary material for: Healthcare inequalities in general practice due to educational level: A retrospective cohort study analysing patients’ presentation and GP response to requests
Source: Eur J Gen Pract. 2026 Apr 14;32(1):2649992. doi: 10.1080/13814788.2026.2649992 (PMC13081339; doi:10.1080/13814788.2026.2649992)
Supplement: Supplemental Material [file IGEN_A_2649992_SM5452.docx]

**Appendix 1: List of chronic diseases (ICPC2-code)**

Cardiovascular

- Rheumatic fever/heart disease (K71)
- Congenital anomaly cardiovascular (K73)
- Ischaemic heart disease with angina (K74)
- Acute myocardial infarction (K75)
- Ischaemic heart disease without angina (K76)
- Heart failure (K77)
- Atrial fibrillation/flutter (K78)
- Paroxysmal tachycardia (K79)
- Cardia arrythmia NOS (K80)
- Heart/arterial murmur (K81)
- Pulmonary heart disease (K82)
- Heart valve disease NOS (K83)
- Other disease of heart (K84)
- Hypertension uncomplicated (K86)
- Hypertension complicated (K87)
- CVA (cerebrovascular accident) (K90)
- Cerebrovascular disease (K91)
- Atherosclerosis/PVD (K92)
- Cardiovascular disease other (K99)

Musculoskeletal

- Congenital anomaly musculoskeletal (L82)
- Neck syndrome (L83)
- Back syndrome without radiating pain (L84)
- Acquired deformity of spine (L85)
- Back syndrome with radiating pain (L86)
- Rheumatoid arthritis (L88)
- Osteoarthrosis of hip (L89)
- Osteoarthrosis of knee (L90)
- Osteoarthrosis other (L91)
- Shoulder syndrome (L92)
- Osteoporosis (L95)
- Acquired deformity of limb (L98)

Mental health

- Chronic alcohol abuse (P15)
- Dementia (P70)
- Organic psychosis, other (P71)
- Schizophrenia (P72)
- Affective psychosis (P73)
- Anxiety disorder (P74)
- Depressive disorder (P76)
- Neurasthenia/surmenage (P78)
- Phobia/compulsive disorder (P79)
- Personality disorder (P80)
- Hyperkinetic disorder (P81)
- Post-traumatic stress disorder (P82)
- Mental retardation (P85)
- Anorexia nervosa/bulimia (P86)
- Psychosis NOS/other (P98)
- Psychological disorders, other (P99)

Eye & Ear

- Congenital anomaly eye other (F81)
- Retinopathy (F83)
- Macular degeneration (F84)
- Cataract (F92)
- Glaucoma (F93)
- Blindness (F94)
- Vertiginous syndrome (H82)
- Presbyacusis (H84)
- Deafness (H86)

(Male and female) urogenital

- Congenital anomaly urinary tract (U85)
- Glomerulonephritis/nephrosis (U88)
- Urinary calculus (U95)
- Urinary disease, other (U99)
- Benign prostatic hypertrophy (Y85)

Respiratory

- Acute/chronic sinusitis (R75)
- Chronic bronchitis (R79)
- Congenital anomaly respiratory (R89)
- Hypertrophy tonsils/adenoids (R90)
- Chronic obstructive pulmonary disease (R95)
- Asthma (R96)
- Allergic rhinitis (R97)

Skin

- Neoplasm skin benign/unspecified (S79)
- Dermatitis seborrhoeic (S86)
- Dermatitis/atopic eczema (S87)
- Dermatitis contact/allergic (S88)
- Psoriasis (S91)

Digestive

- Oesophagus disease (D84)
- Diverticular disease (D92)
- Irritable bowel syndrome (D93)
- Chronic enteritis/ulcerative colitis (D94)
- Liver disease NOS (D97)
- Cholecystitis/cholelithiasis (D98)

Endocrine and metabolic

- Congenital anomaly endocrine/metabolic (T80)
- Goitre (T81)
- Obesity (T82)
- Overweight (T83)
- Hyperthyroidism/thyrotoxicosis (T85)
- Hypothyroidism/myxoedema (T86)
- Diabetes insulin dependent (T89)
- Diabetes non-insulin dependent (T90)
- Gout (T92)
- Lipid disorder (T93)

Neurological

- Poliomyelitis (N70)
- Neurological infection other (N73)
- Congenital anomaly neurological (N85)
- Multiple sclerosis (N86)
- Parkinsonism (N87)
- Epilepsy (N88)
- Migraine (N89)
- Cluster headache (N90)

Blood(forming organs) and lymphatics

- Congenital anomaly blood/lymph other (B79)
- Iron deficiency anaemia (B80)
- Anaemia, vitamin B12/folate deficiency (B81)
- Anaemia other/unspecified (B82)
- Purpura/coagulation defect (B83)
- HIV-infection/AIDS (B90)

General and unspecified

- Secondary effect of trauma (A82)
- Congenital anomaly NOS/multiple (A90)

Infectious

- Tuberculosis (A70)
- Lymphadenitis non-specific (B71)
- Syphilis

Malignancies

- Malignancy NOS (A79)
- Hodgkin’s disease/lymphoma (B72)
- Leukaemia (B73)
- Malignant neoplasm blood other (B74)
- Benign/unspecified neoplasm blood (B75)
- Malignant neoplasm stomach (D74)
- Malignant neoplasm colon/rectum (D75)
- Malignant neoplasm pancreas (D76)
- Malignant neoplasm digestive other / NOS (D77)
- Benign/unspecified neoplasm digestive system (D78)
- Neoplasm of eye/adnexa (F74)
- Neoplasm of ear (H75)
- Neoplasm cardiovascular (K72)
- Malignant neoplasm musculoskeletal (L71)
- Neoplasm benign/unspecified muscuoloskeletal (L97)
- Malignant neoplasm nervous system (N74)
- Benign neoplasm nervous system (N75)
- Neoplasm nervous system unspecified (N76)
- Malignant neoplasm bronchus/lung (R84)
- Malignant neoplasm respiratory, other (R85)
- Benign neoplasm respiratory (R86)
- Neoplasm respiratory unspecified (R92)
- Malignant neoplasm of skin (S77)
- Malignant neoplasm thyroid (T71)
- Benign neoplasm thyroid (T72)
- Neoplasm endocrine other/unspecified (T73)
- Malignant neoplasm of kidney (U75)
- Malignant neoplasm of bladder (U76)
- Malignant neoplasm urinary, other (U77)
- Benign neoplasm urinary tract (U78)
- Neoplasm urinary tract NOS (U79)
- Malignant neoplasm cervix (X75)
- Malignant neoplasm breast female (X76)
- Malignant neoplasm female genital other (X77)
- Benign neoplasm breast female (X79)
- Benign neoplasm female genital (X80)
- Genital neoplasm other/unspecified (X81)
- Malignant neoplasm prostate (Y77)
- Malignant neoplasm male genital other (Y78)
- Benign/unspecified neoplasm male genital (Y79)
